# hsa_circ_0003222 accelerates stemness and progression of non-small cell lung cancer by sponging miR-527

**DOI:** 10.1038/s41419-021-04095-8

**Published:** 2021-08-25

**Authors:** Changhui Li, Jiaqi Zhang, Xiaohua Yang, Cheng Hu, Tianqing Chu, Runbo Zhong, Yinchen Shen, Fang Hu, Feng Pan, Jianlin Xu, Jun Lu, Xiaoxuan Zheng, Hai Zhang, Wei Nie, Baohui Han, Xueyan Zhang

**Affiliations:** 1 Department of Pulmonary, Shanghai Chest Hospital, Shanghai Jiao Tong University, Shanghai, China; 2 Shanghai TCM-Integrated Institute of Vascular Anomalies, Shanghai TCM-Integrated Hospital, Shanghai University of Traditional Chinese Medicine, Shanghai, 200082 China; 3 Central Laboratory, Shanghai Chest Hospital, Shanghai Jiao Tong University, Shanghai, China; 4 Experiment Center for Science and Technology, Shanghai University of Traditional Chinese Medicine, Shanghai, 201203 China

**Keywords:** Cancer microenvironment, Cancer stem cells

## Abstract

The relationship between circular RNA (circRNA) and cancer stem cells (CSCs) is uncertain. We have investigated the combined influence of CSCs, circRNA (hsa_circ_0003222), and immune checkpoint inhibitors in NSCLC progression and therapy resistance. We constructed lung CSCs (LCSCs; PC9 and A549). The effects of hsa_circ_0003222 in vitro were determined by cell counting, colony and sphere formation, and Transwell assays. A tumor xenograft model of metastasis and orthotopic model were built for in vivo analysis. We found that hsa_circ_0003222 was highly expressed in NSCLC tissues and LCSCs. Higher levels of hsa_circ_0003222 were associated with the stage, metastasis, and survival rate of patients with NSCLC. Reduced levels of hsa_circ_0003222 decreased tumor cell proliferation, migration, invasion, stemness-like properties, and chemoresistance. The silencing of hsa_circ_0003222 was found to downregulate PHF21B expression and its downstream, β-catenin by relieving the sponging effect of miR-527. Moreover, silencing hsa_circ_0003222 alleviated NSCLC resistance to anti-programmed cell death-ligand 1 (PD-L1)-based therapy in vivo. Our data demonstrate the significant role of hsa_circ_0003222 in NSCLC cell stemness-like properties. The manipulation of circRNAs in combination with anti-PD-L1 therapy may alleviate NSCLC stemness and progression.

## Introduction

Lung cancer encompasses 11.6% of all diagnosed cancer and contributes to 18.4% of deaths related to cancer worldwide [1–3]. The majority of lung cancers (~85%) have been classified as non-small cell lung carcinoma (NSCLC) by the World Health Organization [4]. NSCLC is notoriously difficult to treat as most patients present at an advanced stage with metastasis and tumor heterogeneity, which poses additional challenges [5–7]. Moreover, drug resistance mediated by genetic and epigenetic alterations often occur in NSCLC [8, 9]. Therefore, several approaches using combination therapies have been used to overcome these barriers, including immune checkpoint inhibitors such as programmed death 1 (PD-1) and programmed death ligand 1 (PD-L1) [10, 11]. Targeting PD-1/PD-L1 (such as pembrolizumab, nicoluzumab, atezolizumab, and durumimab) to block these pathways is to promote the immune response to tumors [12]. To reduce the primary drug resistance rate of tumors, immunotherapy can be combined with chemotherapy or new drugs [13]. Many researched confirmed that PD-L1 is combined with other treatments (such as chemotherapy, EGFR-TKI, and VEGF inhibitors) [14, 15]. However, for cancer stem cells (CSCs), whether PD-L1 combined with other treatments have the same benefits requires further research.

CSCs are increasingly implicated in drug resistance and metastasis in NSCLC and the prognosis in patient-derived samples enriched with CSCs is known to be poor [16, 17]. The self-renewal and plasticity of CSCs allows them to differentiate into different cell types under specific conditions, which may contribute to tumor heterogeneity. For instance, tumor environments are often hypoxic, which induces OCT4 [18]. Several transcription factors including OCT4 have been found to promote pluripotency in somatic cells [19].

MicroRNAs (miRNAs) are viewed as a potential therapy for NSCLC because of their role in regulating genes involved in tumorigenesis and are regularly exploited to investigate the mechanisms of cancer as miRNA mimics, anti-miRNA, and RNA sponges [20]. In particular, miRNAs are involved in the modulation of the immune checkpoints PD-1/PD-L1 [21]. Circular RNAs (circRNAs) are noncoding RNA closed-loop structures that are thought to be involved in the regulation of tumorigenesis [22]. Several circRNAs are implicated as either inhibiting or promoting cell proliferation and migration in NSCLC [23, 24]. The study demonstrated that circRNA hsa-circ-0003222 was found to be differentially regulated in NSCLC [25]. Furthermore, bioinformatics analysis showed that hsa_circ_0003222 is located at chromosome chr12:50848096-50855130 and consists of 7034 bp nucleotides. The mature hsa_circ_00003222 is 317 bp in size and derived from the *LARP4* gene. The *LARP4* gene is involved in mRNA and is known to regulate cell migration and invasion [26, 27]. We discovered that miR-527 is a target of hsa-circ-0003222. Interestingly, miR-527 was found to inhibit the TGF-β/SMAD signaling pathway and suppress epithelial-mesenchymal transition in NSCLC [28]. In addition, PHF21B, a gene that can promote stem-like characteristics in prostate cancer cell lines contains predicted binding sites for miR-527 [29]. Therefore, we aimed to investigate the interactions between hsa-circ-0003222, miR-527, and PHF21B in lung CSCs. In previous research, we found that the synergistic combination of cytotoxic T lymphocytes and the inhibition of PD-L1 could kill CSCs in vitro and alleviate drug resistance in vivo [30]. Therefore, we explored whether hsa-circ-0003222 can influence the regulation of PD-L1 and the subsequent inhibition of CSCs in vitro and in vivo.

## Results

### Expression of hsa_circ_0003222 predicts an unfavorable prognosis in NSCLC patients

**Fig. 1:**
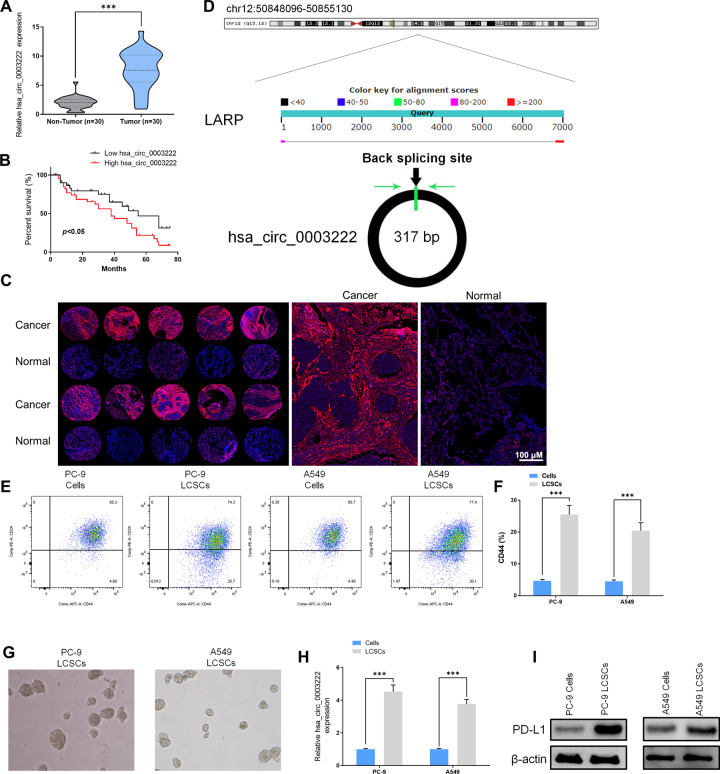
hsa_circ_0003222 predicted a negative prognosis in NSCLC patients. **A** hsa_circ_0003222 level from 30 NSCLC tumor tissues and adjacent non-tumor tissues. **B** Overall survival of NSCLC in relation to hsa_circ_0003222 level. **C** The expression of hsa_circ_0003222 in NSCLC was examined by in situ hybridization on an NSCLC tissue microarray analysis (TMA) (74 cases). **D** *LARP4* gene and hsa_circ_0003222 genomic loci. **E**, **F** Flow cytometry determination of the proportion of PC9 and A549 lung cancer stem cells (LCSCs) expressing CD44. **G** Spheroid formation was assessed in PC9 and A549 LCSCs. **H** RT-qPCR demonstrating hsa_circ_0003222 level in PC9 and A549 or LCSCs. **I** Western blot demonstrating PD-L1 level in PC9 and A549 cells or LCSCs. Data were shown as mean ± SD; ^***^P < 0.001.

To confirm whether there is an association between hsa_circ_0003222 and NSCLC, we determined its relative expression using RT-qPCR in NSCLC tumor tissues and adjacent non-tumor tissues from 30 patients. The clinical characteristics of the patients can be found in Table 1. Levels of hsa_circ_0003222 expression were significantly elevated in tumor tissue compared to the adjacent control tissue (Fig. 1A) and overall survival was influenced by the level of expression (Fig. 1B). The rate of overall survival in NSCLC patients with a lower level of hsa_circ_0003222 expression was higher. Similarly, the expression of hsa_circ_0003222 when analyzed by in situ hybridization on an NSCLC tissue chip (74 cases) was higher than in normal tissue by testing NSCLC samples using a tissue microarray analysis (TMA) (Fig. 1C). These results indicate that a high level of hsa_circ_0003222 expression signifies a negative influence on the overall survival with NSCLC patients. A graphical representation depicting the back splicing of hsa_circ_0003222 from LARP4 can be found in Fig. 1D. Since hsa_circ_00003222 is derived from the LARP4 gene, we evaluated the mRNA level of LARP4. The results showed there is no significant change between tumor and adjacent control tissues (Supplement Fig. 1A). Next, the head-to-tail splicing of endogenous hsa_circ_0003222 was evaluated with convergent and divergent primers. hsa_circ_0003222 could be amplified by the divergent primers in cDNA but not genomic DNA (gDNA) (Supplement Fig. 1B). Resistance to digestion with RNase R exonuclease also confirmed that hsa_circ_0003222 harbored a circRNA structure (Supplement Fig. 1C).

**Table 1:** Relationship between hsa_circ_0003222 expression and the clinical-pathological characteristics in of NSCLC patients (*n* = 30).

Clinic pathological features		No. of cases	hsa_circ_0003222 (*n*, %)		*p* value
			Low	High	
Gender	Male	18	8	10	>0.05
	Female	12	5	7	
Age	≤60	17	7	10	>0.05
	>60	13	6	7	
Smoking status	Yes	19	9	10	>0.05
	No	11	4	7	
Tumor size	≤3 cm	14	9	5	<0.05
	>3 cm	16	4	12	
Tumor stage	I/II	13	8	5	<0.05
	III/IV	17	5	12	
Lymph node metastasis	Yes	17	4	13	<0.05
	No	13	9	4	
Histological type	Squamous cell carcinoma	11	5	6	>0.05
	Adenocarcinomas	19	8	11	
Differentiation	Well and moderate	15	10	5	<0.05
	Poor	15	3	12

The stem-like characteristics of LCSCs generated from PC9 and A549 cell lines were confirmed by assessing the expression of CD44, a cell surface marker for CSC populations. Flow cytometry indicated that the LCSCs generated from PC9 and A549 cell lines had a significantly higher expression of CD44 than the controls (*P* < 0.001, Fig. 1E, F). Spheroid formation verified the generation of LCSCs (Fig. 1G). Moreover, the expression of hsa-circ-0003222 was assessed with RT-qPCR and found to be significantly higher in the LCSCs of both cell lines (*P* < 0.001, Fig. 1H). PD-L1 expression was also elevated in the LCSCs of PC9 and A549 cell lines according to western blot analysis (Fig. 1I). The high expression of hsa_circ_0003222 and PD-L1 expression in LCSCs may contribute to the poor prognosis of patients in NSCLC.

### NSCLC cell proliferation and stemness are suppressed by the downregulation of hsa_circ_0003222 in vitro

**Fig. 2:**
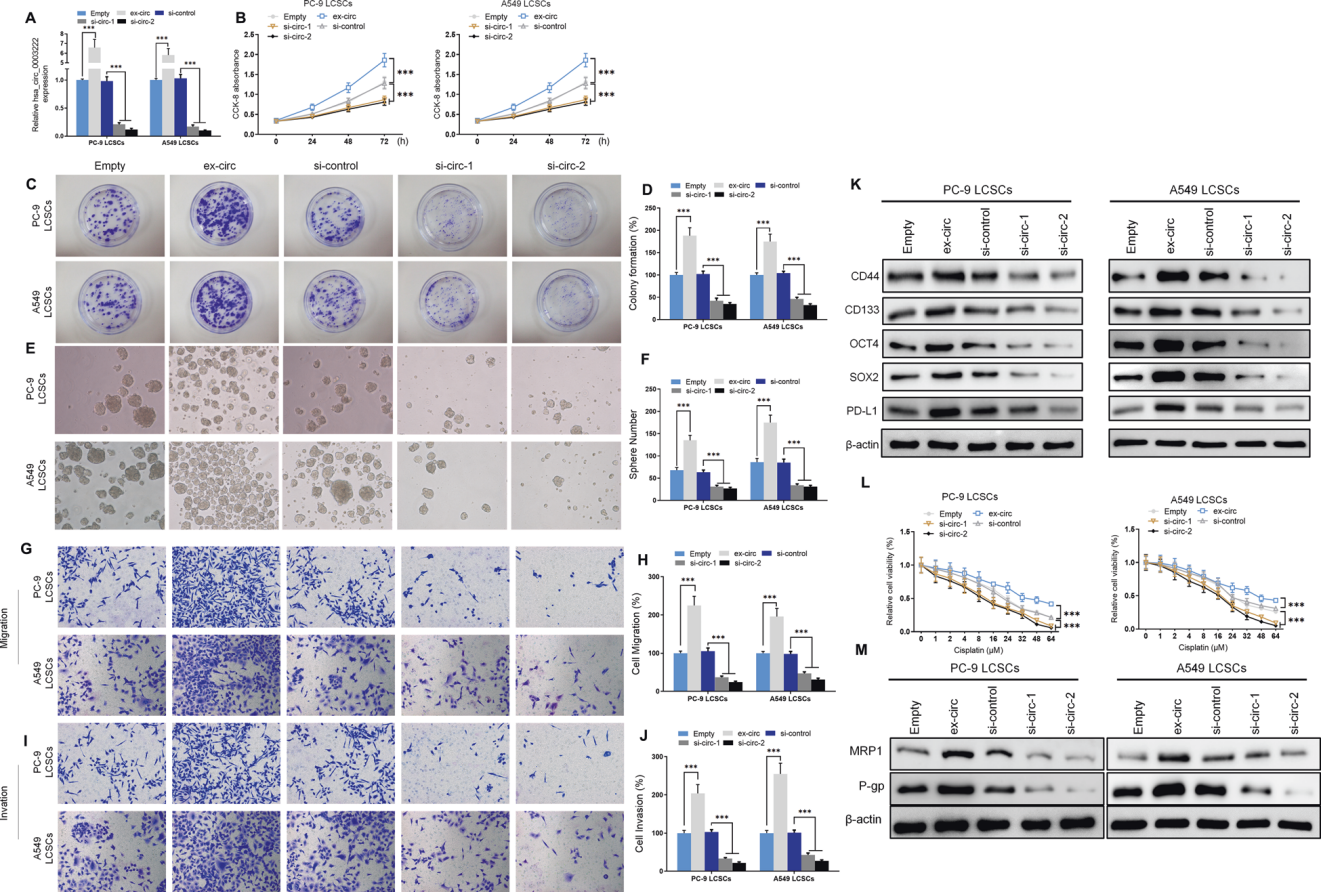
Silencing of hsa_circ_0003222 blocked NSCLC cell proliferation and stemness. **A** RT-qPCR demonstrating hsa_circ_0003222 level in PC9 and A549 lung cancer stem cells (LCSCs). **B** CCK8 assays presented cell proliferation at different times. **C**, **D** Colony formation assay showing proliferation in LCSCs. **E**, **F** Spheroid formation was assessed in LCSCs. **G**–**J** Cells migration and invasion were detected by Transwell assay. **K** Western blot demonstrating CD44, CD133, OCT4, SOX2, and PD-L1 levels in LCSCs. **L** CCK8 assays presented cell proliferation in response to different concentrations of cisplatin. **M** Western blot demonstrating MRP1 and P-gp levels in LCSCs. Data were shown as mean ± SD; ^***^P < 0.001.

To obtain further evidence of hsa_circ_0003222 involvement in the stemness of cells, we measured the influence of manipulating its expression on cell proliferation and colony and spheroid formation. We transfected PC9 and A549 LCSCs with hsa_circ_0003222 expression and inhibition vectors and measured hsa_circ_0003222 levels with RT-qPCR to confirm over and under expression (Fig. 2A). Cell proliferation was significantly higher when hsa_circ_0003222 was overexpressed and lower when the expression was inhibited (Fig. 2B). Similar results were obtained from colony and sphere formation (Fig. 2C–F). To assess whether manipulating the expression of hsa_circ_0003222 also influenced cell migration and drug resistance, we conducted Transwell assays (Fig. 2G–J) and determined levels of stem cell-associated proteins by western blotting (Fig. 2K). We found that the migration and invasion of the LCSCs were significantly increased when hsa_circ_0003222 was overexpressed (*P* < 0.001) whereas migration and invasion were significantly reduced when hsa_circ_0003222 was inhibited. (*P* < 0.001). Likewise, the expression levels of stem cell-associated proteins, CD44, CD133, OCT4, SOX2, and PD-L1, in PC9 and A549 LCSCs appeared to be elevated when hsa_circ_0003222 was overexpressed but was lowered when hsa_circ_0003222 expression was inhibited. Moreover, cell viability in response to different concentrations of cisplatin was increased significantly (*P* < 0.001) when hsa_circ_0003222 is overexpressed and reduced significantly when it is inhibited (*P* < 0.001) (Fig. 2L). Furthermore, proteins associated with drug resistance MRP1 and P-gp were upregulated in PC9 and A549 LCSCs when hsa_circ_0003222 expression increases but are less detectable when hsa_circ_0003222 expression decreases (Fig. 2M). Besides, since PD-L1 upregulation was dependent on IFN-γ secreting, our results confirmed that silencing of hsa_circ_0003222 suppressed the upregulation of PD-L1 induced by IFN-γ (5 ng/mL).

In addition, we transfected SW900 LCSCs, a squamous carcinoma cell line with has_circ_0003222 expression and inhibition vectors and measured has_circ_0003222 levels with RT-qPCR to confirm over and under expression (Supplement Fig. 2A). Cell proliferation was significantly increased when has_circ_0003222 was overexpressed, but decreased when has_circ_0003222 was inhibited (Supplement Fig. 2B). Similar results were obtained for colony formation and sphere formation (Supplement Fig. 2C–F). Furthermore, we conducted Transwell assays to detect the role of hsa_circ_0003222 to affected cell migration and drug resistance (Supplement Fig. 2G–H) and determined levels of stem cell-associated proteins by western blotting (Supplement Fig. 2I). When has_circ_0003222 was overexpressed, SW900 migration and invasion were significantly increased. And the expression levels of stem cell-related proteins CD44, CD133, OCT4, SOX2, and PD-L1 in SW900 LCSCs were increased. In addition, the expression level of PHF21B was also increased (Supplement Fig. 2J).

These results combined, suggest hsa_circ_0003222 may plan a significant regulatory mechanism in the generation of stem cells, tumorigenesis, and drug resistance in lung cancer.

### MiR-527 is suppressed by hsa_circ_0003222 in PC9 and A549 LCSCs

**Fig. 3:**
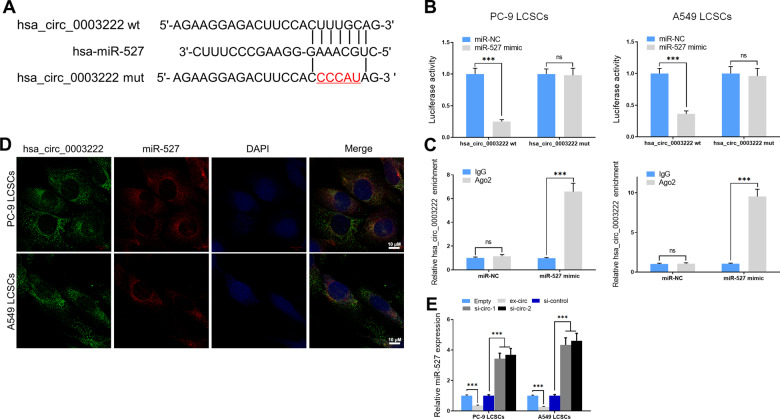
hsa_circ_0003222 sponged miR-527 in PC9 and A549 lung cancer stem cells (LCSCs). **A** The predicted binding sites of miR-527 in the hsa_circ_0003222. The mutated (Mut) version of hsa_circ_0003222 is presented. **B** Relative luciferase activity was examed 48 h after transfection with miR-527 mimic/normal control (NC) or with the hsa_circ_0003222 wild-type/Mut in PC9 and A549 LCSCs. **C** Anti-AGO2 RIP was displayed in PC9 and A549 LCSCs, followed by RT-PCR to access hsa_circ_0003222. **D** FISH assays were performed to evaluate hsa_circ_0003222 and miR-527 location. **E** RT-qPCR demonstrating miR-527 level in LCSCs. Data were presented as mean ± SD; ^***^P < 0.001.

To determine the possible targets of hsa_circ_0003222, miRNAs with differentially expressed between control LCSCs and those with hsa_circ_0003222 inhibited were examined (Table 2). We found that hsa-miR-527 was highly expressed when hsa_circ_0003222 was inhibited and was predicted to be a possible target by CircInteractome (https://circinteractome.nia.nih.gov). We mutated the predicted binding sites of miR-527 in hsa_circ_0003222 (Fig. 3A) and measured relative luciferase activity after transfection with miR-527 to confirm an interaction (Fig. 3B). To further confirm this interaction, we also performed anti-AGO2 RIP in PC9 and A549 LCSCs transfected with miR-527 and detected the presence of hsa_circ_0003222 in the immunoprecipitation assay by RT-qPCR (Fig. 3C). Using a FISH assay we determined that hsa_circ_0003222 and miR-527 were co-located in LCSCs (Fig. 3D) and RT-qPCR indicated that the expression of miR-527 in PC9 and A549 LCSCs was significantly elevated when hsa_circ_0003222 is inhibited by interference RNA (Fig. 3E). Overall, these results indicate that the miR-527 is targeted by hsa_circ_0003222 in LCSCs.

**Table 2:** Upregulated miRNA in the si-circ2 PC9 cells compared to si-control PC9 cells.

miRNAs	Fold-change	*p* value
hsa-miR-1298-5p	7.12174537	0.008377505
hsa-miR-1257	6.21629274	0.045909597
hsa-miR-509-3-5p	4.849981574	0.008005709
hsa-miR-569	4.265466081	0.00907297
hsa-miR-1290	3.533853563	0.004906548
hsa-miR-139-3p	3.184911439	0.034220671
hsa-miR-210-5p	2.999914028	0.043007773
hsa-miR-3662	2.520338371	0.03386189
hsa-miR-527	2.42355494	0.007033471

### Inhibition of miR-527 reversed the suppressive effect of hsa_circ_0003222 silencing on NSCLC cell proliferation and stemness in vitro

**Fig. 4:**
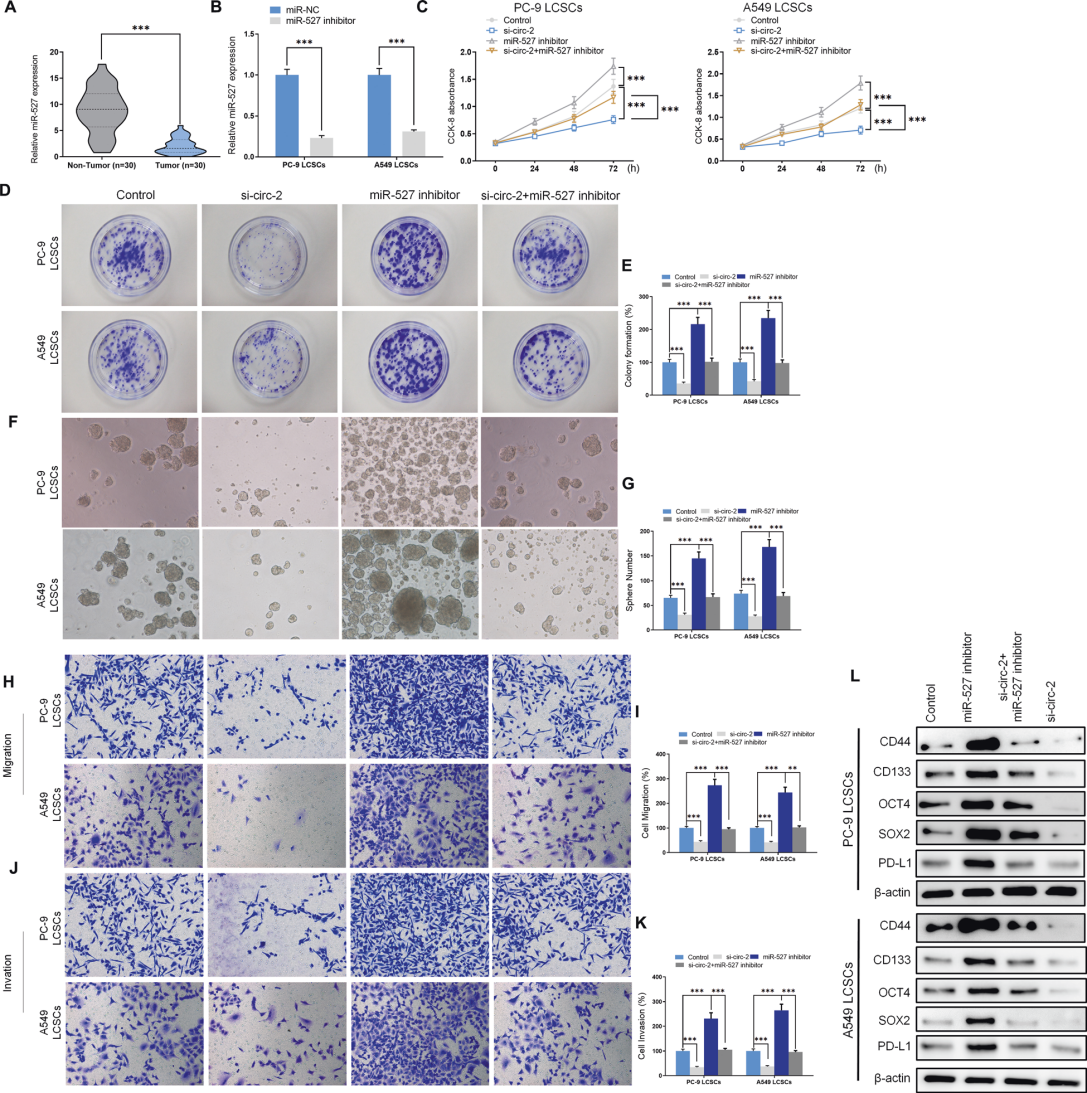
Inhibition of miR-527 reversed the block effectiveness of hsa_circ_0001421 suppression on NSCLC cell proliferation and stemness in vitro. **A** RT-qPCR demonstrating miR-527 level from 30 NSCLC tumor tissues and adjacent non-tumor tissues. **B** RT-qPCR demonstrating miR-527 level in LCSCs. **C** CCK8 assays presented cell proliferation at different time points. **D**, **E** Colony formation presented proliferation in LCSCs. **F**, **G** Spheroid formation was assessed in PC9 and A549 lung cancer stem cells (LCSCs). **H**–**K** Transwell assays presented cells migration and invasion. **L** Western blot demonstrating CD44, CD133, OCT4, SOX2, and PD-L1 levels in PC9 and A549 LCSCs. Data were shown as mean ± SD; ^**^P < 0.01, ^***^P < 0.001.

To gain a greater understanding of the role played by miR-527 in NSCLC, we measured its expression in 30 NSCLC tumor and paratumor tissues and found that, in contrast to hsa_circ_0003222, miR-527 expression levels were lower in tumor tissue (Fig. 4A). The expression of miR-527 could be reduced by an inhibitor in PC9 and A549 LCSCs (Fig. 4B) and this led to a significantly higher level of proliferation compared to the control and in LCSCs silenced by hsa_circ_0003222 (*P* < 0.001, Fig. 4C). In fact, proliferation was significantly lower in stem cells with just hsa_circ_0003222 inhibited than with both hsa_circ_0003222 and miR-527 inhibited (*P* < 0.001). Similarly, the inhibition of miR-527 significantly increased colony and spheroid formation in both PC9 (*P* < 0.001) and A549 (*P* < 0.001) LCSCs and restored the colony and spheroid formation that were reduced by the inhibition of hsa_circ_0003222 (Fig. 4D–G). Cell migration and an invasion followed a similar pattern and were also increased by the inhibition of miR-527 (Fig. 4H–K). Likewise, the expression of stem cell-related proteins was elevated in PC9 and A549 LCSCs when miR-527 is inhibited (Fig. 4L). Overall, these data indicate that the suppressive result of hsa_circ_0003222 abolishing on NSCLC cell proliferation and stemness can be reversed to some extent by inhibiting miR-527. This suggests that miR-527 expression can prevent cells from adopting stem-like characteristics.

### miR-527 involvement in LCSCs is mediated by the modulation of PHF21B

**Fig. 5:**
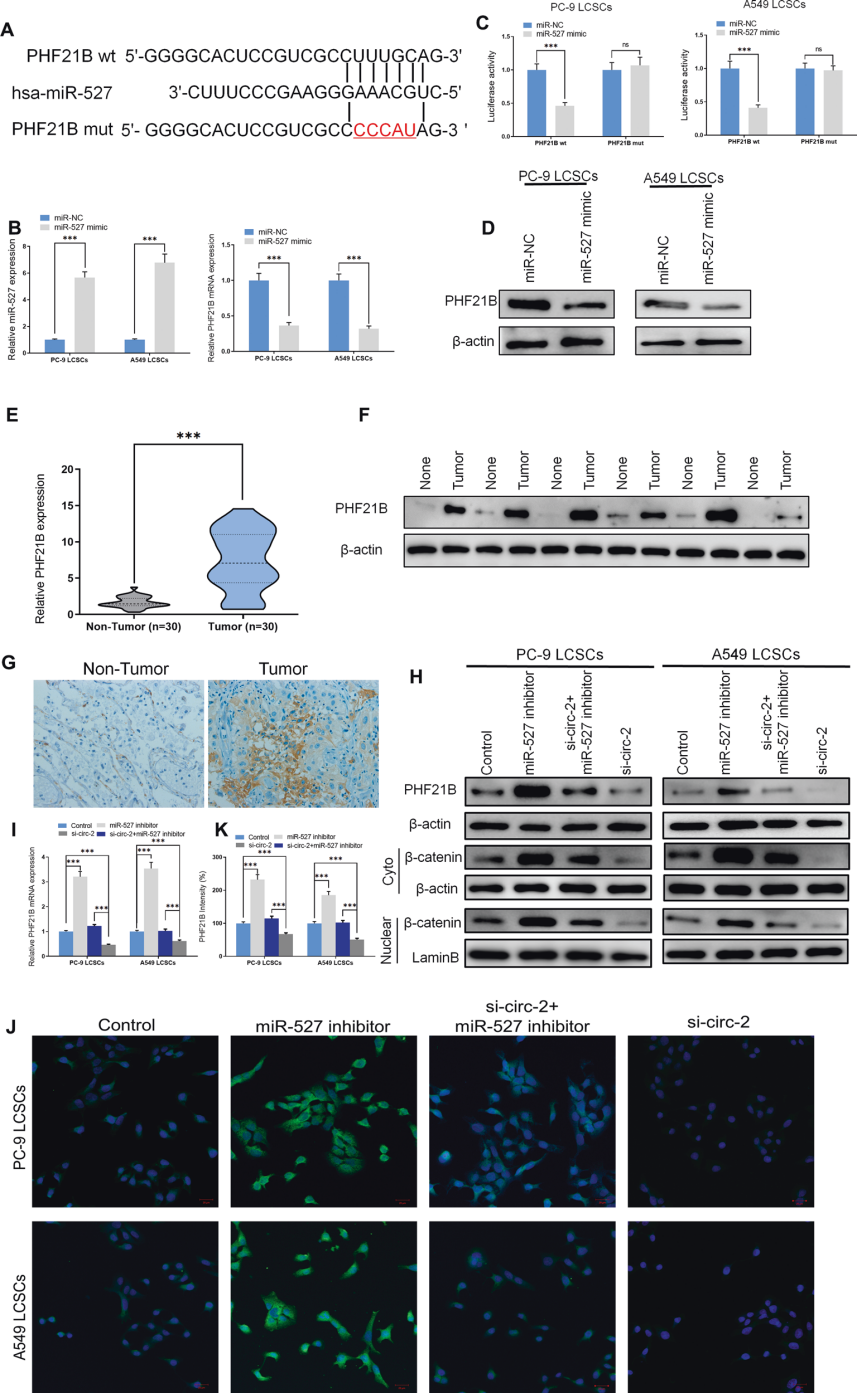
miR-527 involvement in lung cancer stem cells (LCSCs) is facilitated by the regulation of PHF21B. **A** The predicted binding sites of miR-527 in PHF21B. The mutated (Mut) version of PHF21B is also presented. **B** Relative luciferase activity was measured 48 h after transfection in PC9 and A549 LCSCs. **C** RT-qPCR demonstrating miR-527 and PHF21B levels in LCSCs. **D** Western blot demonstrating PHF21B level in PC9 and A549 LCSCs. **E** RT-qPCR demonstrating PHF21B level from 30 NSCLC tumor tissues and adjacent non-tumor tissues. **F** Western blot demonstrating PHF21B level in 30 NSCLC tumor tissues and adjacent non-tumor tissues. **G** Immunohistochemistry of PHF21B level in 30 NSCLC tumor tissues and adjacent non-tumor tissues. **H** RT-qPCR demonstrating miR-527 and PHF21B levels in PC9 and A549 LCSCs. **I** Western blot demonstrating PHF21B and β-catenin levels in PC9 and A549 LCSCs. **J**, **K** Immunofluorescence demonstrating PHF21B level in PC9 and A549 LCSCs. Data were shown as mean ± SD; ^***^P < 0.001.

We located potential binding sites of miR-527 in PHF21B, a gene that has been found to promote cancer stemness (Fig. 5A) [29]. We confirmed that an interaction exists between miR-527 and PHF21B by conducting a luciferase assay (Fig. 5B). The overexpression of miR-527 was found to significantly reduce the RNA expression and protein levels of PHF21B (Fig. 5C, D). Further characterization of PHF21B, revealed that it was significantly upregulated in NSCLC tumor tissues (Fig. 5E–G). The inhibition of miR-527 in PC9 and A549 LCSCs upregulated the expression of PHF21B whereas the inhibition of hsa_circ_0003222 downregulated the expression of PHF21B and these data were well-supported by immunofluorescence studies (Fig. 5H–K). Therefore, PHF21B seems to function in tumor progression in NSCLC and is regulated by miR-527. Besides, a previous study has reported that PHF21B promoted the activation of the Wnt/β-catenin pathway and enhanced cell stemness [29]. Our results confirmed that the inhibition of hsa_circ_0003222 impeded the nuclear transcription of β-catenin while miR-527 reversed it (Fig. 5H).

### Silencing hsa_circ_0003222 alleviated NSCLC resistance to anti-PD-L1 in vivo

**Fig. 6:**
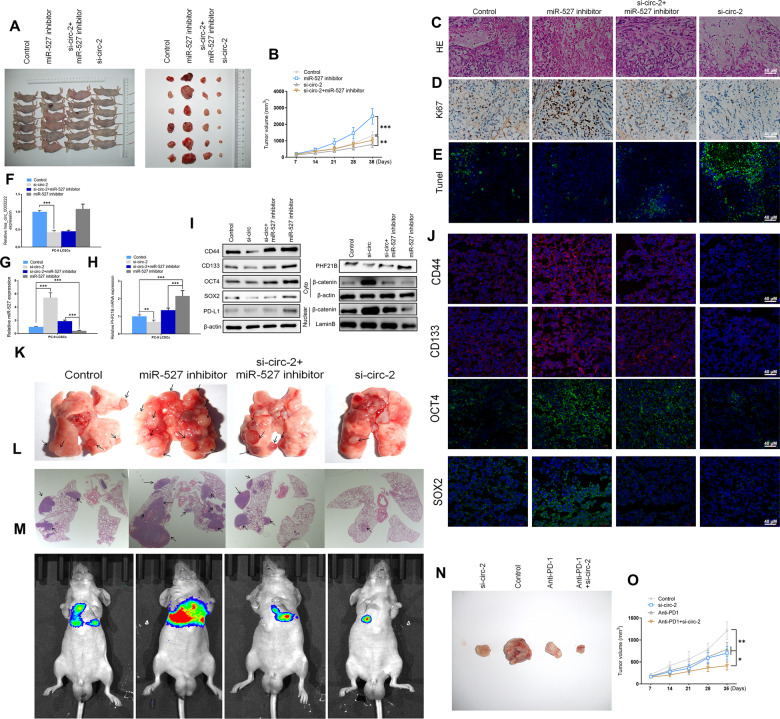
Influence of hsa_circ_0003222 and miR-527 in vivo. **A** Representative images of xenograft tumors isolated from nude mice in the different groups. **B** Tumor sizes in different groups. **C** HE staining in xenograft tumors tissue. **D** Immunohistochemistry of Ki-67 in xenograft tumors tissue. **E** TUNEL assay in xenograft tumors tissue. **F**–**H** RT-qPCR demonstrating hsa_circ_0003222, miR-527, and PHF21B levels in xenograft tumors tissue. **I** Western blot demonstrating CD44, CD133, OCT4, SOX2, PD-L1, PHF21B, and β-catenin levels in xenograft tumors tissue. **J** Immunofluorescence showing the expression of CD44, CD133, OCT4, and SOX2 in xenograft tumors tissue. **K** Representative images of orthotopic model tumors isolated from nude mice in the different groups. **L** Representative HE staining of orthotopic model tumors isolated from nude mice in the different groups. **M** Live imaging demonstrating hsa_circ_0003222 and miR-527 effects on the metastasis of PC9 LCSCs 30 days after intravenous tail injection. **N** Representative images of xenograft tumors isolated from nude mice in the different groups after treatment with anti-PD-L1. **O** Tumor sizes in the various groups. Data were presented as mean ± SD; *P < 0.05, ^**^P < 0.01.

We next assessed whether the results obtained in vitro could translate into a xenograft model of NSCLC tumors. NSCLCs inhibiting miR-527, hsa_circ_0003222, or both together were injected subcutaneously into mice. The inhibition of miR-527 resulted in the highest tumor volume (*P* < 0.001) whereas the inhibition of hsa_circ_0003222 resulted in a tumor volume minor than control (*P* < 0.01) (Fig. 6A, B). Representative HE staining in xenograft tumor tissue (Fig. 6C), immunohistochemistry of Ki-67 (Fig. 6D), and a TUNEL assay (Fig. 6E) all indicate that in xenograft tumor tissue, cell proliferation is increased when miR-527 is inhibited and apoptosis is increased when hsa_circ_0003222 is inhibited. The levels of hsa_circ_0003222 and PHF21B are meaningfully increased in tumor tissue when miR-527 is inhibited (Fig. 6F–H). The levels of stem cell-associated proteins measured in tumor tissue by western blotting and immunofluorescence were reduced by hsa_circ_0003222 inhibition but elevated by miR-527 inhibition (Fig. 6I, J). Orthotopic models of tumors were also reproduced in nude mice in the different treatments (Fig. 6K, L). Live imaging shows that hsa_circ_0003222 inhibition prevents metastasis whereas miR-527 inhibition increases metastasis in PC9 LCSCs after 30 days (Fig. 6M). Moreover, treatment with a combination of anti-PD-L1 and hsa_circ_0003222 inhibition was found to significantly reduce tumor volume after 35 days (Fig. 6N, O). Our data revealed that the inhibition of hsa_circ_0003222 can alleviate NSCLC resistance to anti-PD-L1 in vivo.

## Discussion

Despite current advances in the therapy of cancer with immune checkpoint inhibitors, NSCLC remains a leading cause of cancer fatality, partly owing to drug resistance and metastasis caused by stem cells [31, 32]. To overcome these challenges various approaches have been adopted to enhance the susceptibility of NSCLC to therapy [33]. In this work, we have concentrated on suppressing the role of LCSCs in drug resistance and metastasis by exploiting the regulatory characteristics of circRNA.

In our study, we searched for circRNA that may be potential regulators of LCSCs and subsequent metastasis and drug resistance in NSCLC. Using immunochemistry, we found that the level of hsa_circ_0003222 was upregulated in the tumor tissue of patients with NSCLC and that it could predict an unfavorable prognosis. Hsa_circ_0003222 is derived from LARP4, a gene associated with cell division and RNA stability [26, 27]. Mutations in LARP4 are frequently associated with cancer [27]. More recently, circular LARP4 was found to suppress metastasis in NSCLC by upregulating its predicted protein SMAD7 [34]. SMAD7 is thought to regulate the progression of metastasis through the inhibition of transforming growth factor β (TGFβ) receptor signaling, which plays a major role in epithelial-mesenchymal cell conversion [35]. Whether hsa_circ_0003222 expression has an adverse effect on SMAD7 and TGFβ receptor signaling and the relationship between hsa_circ_0003222 and circular LARP4 requires further study.

Several circRNAs have been found to control cell cycle events and gene expression in NSCLC by sponging miRNA [36–38]. In some instances, circRNA have acted as tumor suppressors, for instance, Hsa_circ_0002483 was found to inhibit NSCLC progression and enhance Taxol sensitivity by sponging targeting miR-182-5p [39]. However, circRNA also contributes to the progression of cancer. In NSCLC, circFGFR1 was found to promote cancer progression and anti-PD-L1 resistance by sponging miR-381-3p [38]. Similar to in our study, the circRNA was associated with poor prognosis in patients through the indirect upregulation of a target gene, CXCR4, which is involved in the promotion of cell cycle progression.

In this study, we found that both the expression of hsa_circ_0003222 and that of PD-L1 were upregulated in LCSCs. The upregulation of PD-L1 signifies a poor prognosis in NSCLC because it negatively regulates levels of CD4+ and CD8+ tumor-infiltrating T lymphocytes, which are associated with a better prognosis [12]. Therefore, we manipulated levels of hsa_circ_0003222 expression to determine whether this could influence PD-L1 levels. When hsa_circ_0003222 was overexpressed levels of PD-L1 were also upregulated as were the levels of proteins associated with stemness, CD44, CD133, OCT4, and SOX2, and the drug resistance proteins MRP1 and P-gp. Moreover, the upregulation of hsa_circ_0003222 increased colony and sphere formation, resistance to cisplatin, and the migration and invasiveness of LCSCs. Our results indicate that hsa_circ_0003222 contributes to the progression of NSCLC.

We then focused our attention on finding the potential targets of hsa_circ_0003222. Small sequencing and online tools predicted that miR-527 could be a potential candidate. It has been reported that miR-527 is associated with the inhibition of the TGF-β/SMAD signaling pathway through the regulation of SULF2 to suppress epithelial-mesenchymal transition in NSCLC [28]. We found that levels of miR-527 were lower in the tumor tissue of NSCLC patients than in adjacent non-tumor tissue. Our studies confirmed that miR-527 had a suppressive role in NSCLC. In addition, we found that miR-527 is suppressed by hsa_circ_0003222 and that the inhibition of miR-527 reversed the suppressive effect of hsa_circ_0003222 silencing on NSCLC cell proliferation and stemness in vitro and in vivo. We discovered that PHF21B was the regulatory target of miR-527 and that PHF21B is significantly upregulated in the tumor tissues of patients with NSCLC. Moreover, the overexpression of miR-527 was found to significantly reduce the RNA expression and protein levels of PHF21B in LCSCs. In prostate cancer, PHF21B overexpression has been found to promote CSC-like traits cells by activating the Wnt/β-catenin signaling pathway [29]. However, PHF21B has also been found to act as a tumor suppressor in head and neck squamous cell carcinomas [40]. A higher expression of PHF21B was found to correlate with DNA methylation, suggesting that epigenetic mechanisms may control its regulation in cancer [40].

The results we obtained in vitro were replicated convincingly in vivo in a murine xenograft model. Cell proliferation and tumor volume were increased when miR-527 is inhibited whereas apoptosis is increased when hsa_circ_0003222 is inhibited. An orthotopic model of tumors in nude mice indicated that hsa_circ_0003222 inhibition prevents metastasis and that treatment with a combination of anti-PD-L1 and hsa_circ_0003222 inhibition was found to significantly reduce tumor volume after 35 days. These results imply that the inhibition of PD-L1 and hsa_circ_0003222 could be used in combination to alleviate metastasis and drug resistance in NSCLC.

To conclude, hsa_circ_0003222 accelerates stemness and the progression of NSCLC by sponging miR-527. MiR-527 expression levels were lower in tumor tissue and inhibit stemness and the progression of NSCLC. The inhibition of hsa_circ_0003222 may alleviate NSCLC resistance to anti-PD-L1. These findings illustrate the importance of circRNAs in the stemness and progression of NSCLC and in PD-L1 therapy.

## Materials and methods

### Tissue samples

We collected 30 cases of fresh NSCLC tumor and paired paratumor from patients who agreed to the informed consent at the Shanghai Chest Hospital of Shanghai Jiao Tong University, China. None of these patients received chemotherapy or radiotherapy before collecting of tissue samples, which were then immediately snap-freezing and kept at −80 °C. This study was permitted by the Ethics Committee of Shanghai Chest Hospital at Shanghai Jiao Tong University (ks(y)21167). All patients agreed that their lung tissues and information were used for research and signed written informed consent before the collection of lung tissues and information.

### Cell culture

We purchased Human lung cancer cells (PC9 and A549 cells) from the Cell Bank of the Chinese Academy of Sciences (Shanghai, China). Cells in 90% Dulbecco modified Eagle’s medium (DMEM; Gibco, Grand Island, NY) were in a 37 °C humidified incubator with 5% carbon dioxide.

### Lung cancer stem‐like cell (LCSC) construction

We used repeated increase cisplatin therapy and sphere formation methods to construct the PC9 and A549 LCSC models, which were also described in our previous study [30]. In short, we treated cells with cisplatin in escalating concentration (1 to 10 μmol/L). We cultivated cells in serum‐free stem cells medium to build the spheres.

### Cell transfection

Cells were transfected with an appropriate amount of vector by using Lipofectamine 2000 (Invitrogen, MA, USA) and then cultured for 48 h on the basis of the manufacturer’s protocol.

### Vector construction

The full-length cDNA of hsa_circ_0003222 was synthesized by GeneChem (Shanghai, China) and then cloned into the circRNA vector (GV535, purchased from GeneChem). Two siRNAs against hsa_circ_0003222, shown in Supplementary Table 1, were also synthesized by GeneChem (Shanghai, China). The expression efficiency was examined using qPCR in cells transfected with vector or siRNA. miR-527 inhibitor and control (miR-NC) were all created by Hanbio (Shanghai, China), which are presented in Supplementary Table 1.

### Animal studies

The orthotopic assay was consistent with what was mentioned in our previous study [30]. We prepared the PC9 LCSC suspension at a density of 1 × 10^7^ ml and mixed 50 μl cells suspension and 50 ml Matrigel and subsequently injected into the left lung of the mice through the chest wall at depth of 3 mm. Magnetic resonance imaging (MRI) examinations were used 1 week later to exam tumor formation. Each group contained six mice.

We fostered and handled all experimental animals approved by the Animal Care Committee of Shanghai Chest Hospital of Shanghai Jiao Tong University.

### Statistical analysis

All data were statistically examined by GraphPad 7.0. *T*-test was performed between two independent groups; one-way ANOVA test was applied among various groups; Kaplan–Meier curves and the log-rank test were used to analyze the survival rate of patients. *p* < 0.05 was considered a statistical significance.

More detailed materials and methods can be found in the Supplementary Methods.

## Supplementary information

Supplemental Information

supplement figure 1

supplement figure 2
