# Supplementary material for: hsa_circ_0003222 accelerates stemness and progression of non-small cell lung cancer by sponging miR-527
Source: Cell Death Dis. 2021 Aug 25;12(9):807. doi: 10.1038/s41419-021-04095-8 (PMC8387484; doi:10.1038/s41419-021-04095-8)
Supplement: Supplementary file 1 — Supplemental Information [file 41419_2021_4095_MOESM1_ESM.docx]

**Supplement Figure 1.** Identification and distribution of has-circ-0003222.


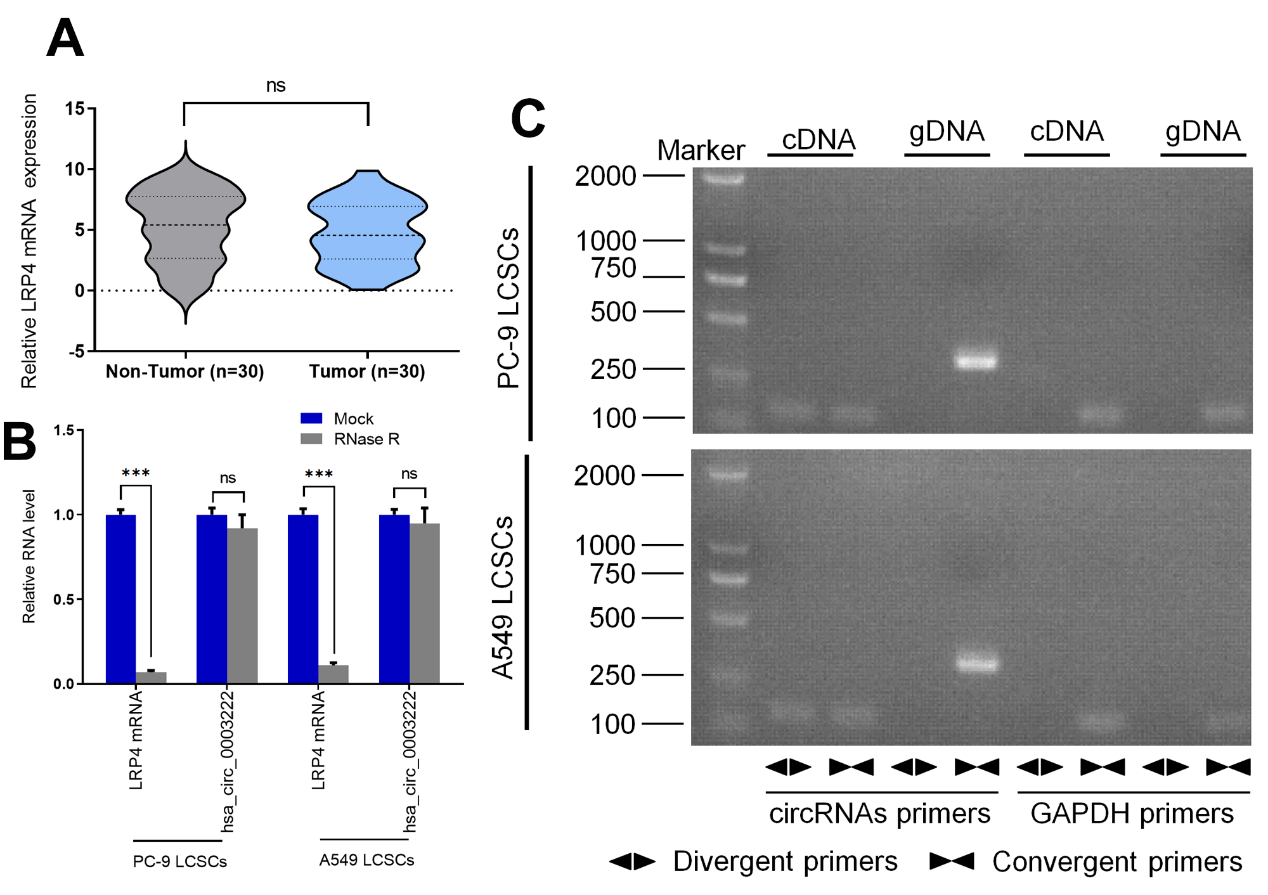


Supplement Figure 1. A. LRP4 mRNA level from 30 NSCLC tumor tissues and adjacent non-tumor tissues. B. The existence of hsa_circ_0003222 was validated in LCSCs by qRT-PCR. Divergent primers amplified hsa_circ_0003222 in cDNA but not genomic DNA (gDNA). GAPDH was used as negative control. C. The relative RNA levels were analyzed by qRT-PCR in LCSCs treated with or without RNase R.

**Supplement Figure 2.** The expression of has-circ-0003222 was also increased in SW900.


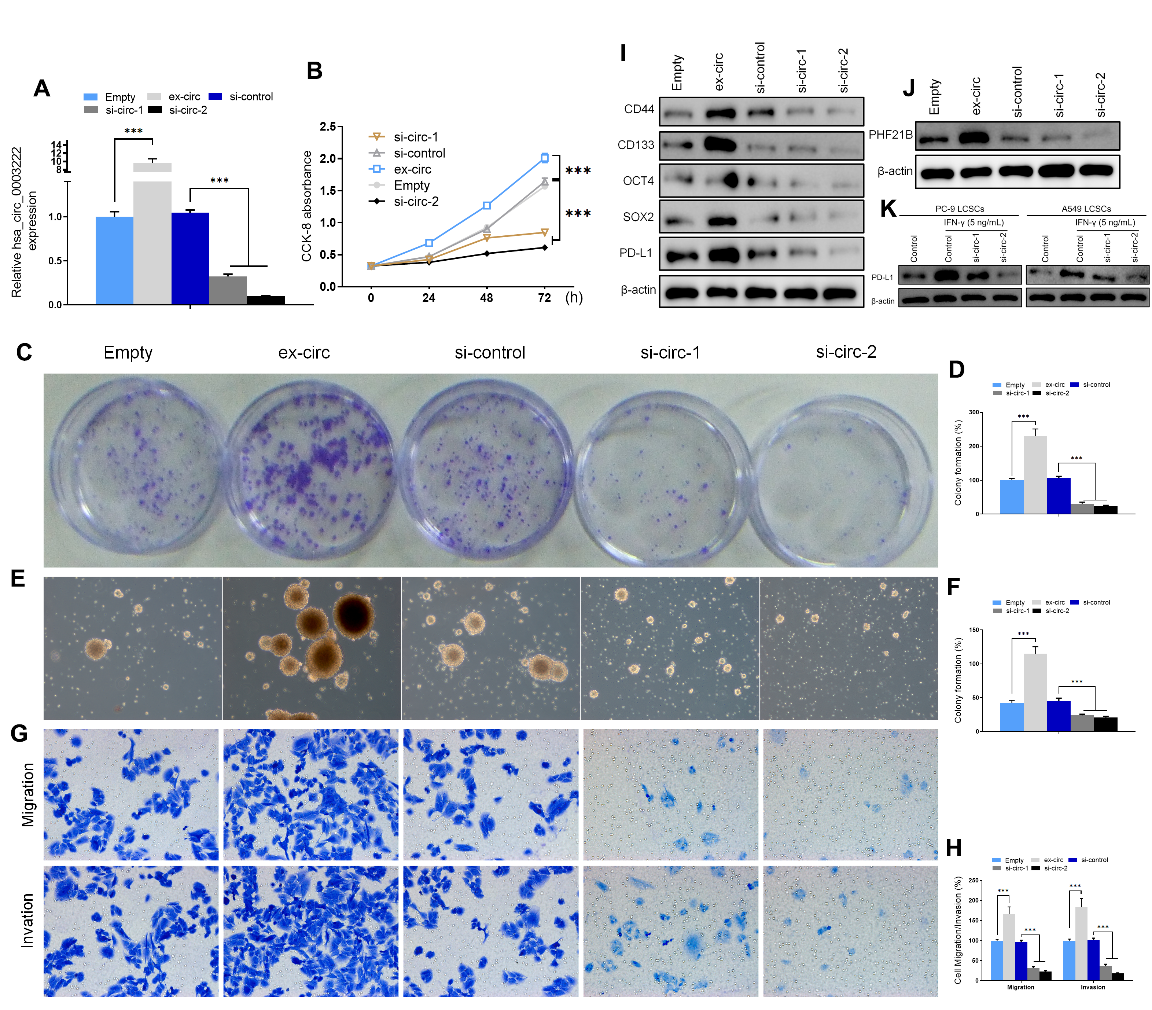


Supplement Figure 2. A. RT-qPCR detection showing the expression of hsa_circ_0003222 in SW900 LCSCs. B. CCK8 assays presented cell proliferation at different times. C and D. Colony formation assay presented proliferation in SW900 LCSCs. E and F. Spheroid formation was assessed in SW900 LCSCs. G and H. Cells migration and invasion were detected by Transwell assay. I. Western blot demonstrating CD44, CD133, OCT4, SOX2, and PD-L1 levels in SW900 LCSCs. J. The expression level of PHF21B increased when has_circ_0003222 was overexpressed. K. The expression level of PD-L1 was evaluated after treating with IFN-γ (5 ng/mL) for 24 hours. Data are presented as mean ± SD; ***P<0.001.

**Table S1. siRNA sequences in the research**

| **Name** | **SiRNA sequence** |
| --- | --- |
| **Hsa_circ_0003222 siRNA1** | GGGCAGGCTCCCTTTCCCAAT |
| **Hsa_circ_0003222 siRNA2** | GGGGCAGGCTCCCTTTCCCAA |
| **Negative control siRNA** | CCUACAUCCCGAUCGAUGAUGUUGA |
| **MiR-527 inhibitor** | GACAGGTTATCAACGAAACTTCT |
| **Inhibitor negative control** | UCACAACCUCCUAGAAAGAGUAGA |

**Methods**

**Fluorescence in situ hybridization (FISH)**

Specific probes to hsa_circ_0003222 were prepared by Geneseed Biotech (Guangzhou, China). Signals were detected by Cy3-conjugated anti-digoxin and FITC-conjugated anti-biotin antibodies (Jackson Immuno Research Inc., West Grove, PA). Nuclei were counterstained with 4,6-diamidino-2-phenylindole (DAPI). Finally, images were obtained on a Zeiss LSM 700 confocal microscope (Carl Zeiss, Oberkochen, Germany).

**Library preparation for small RNA sequencing**

Sample was used to build a small RNA library as former describe[[1](#_ENREF_1), [2](#_ENREF_2)]. Sequencing libraries were generated using NEBNext Multiplex Small RNA Library Prep Set for Illumina (NEB, Ipswich, MA) following the manufacturer’s recommendations. Library quality was assessed on an Agilent Bioanalyzer 2100 system using DNA High Sensitivity Chips.

**Total RNA isolation and RT-qPCR assay**

Total RNA was isolated using TRIzol (Invitrogen). Primers for hsa_circ_0003222, PHF21B, and miR-527 were attained from GenePharma (Shanghai, China). *GAPDH* was used as the reference gene for circRNAs and mRNAs. U6 was used as an internal control for the level of miRNA expression. Gene expression was quantified using the 2^−ΔΔCt^ method as former describe[[3](#_ENREF_3)].

**Protein extraction and western blot analysis**

The lung tissue and LCSCs was lysed using RIPA buffer and the protein concentration was determined using a BCA protein assay kit (Thermo Fisher). Western blot analysis were described in our previous study[[3](#_ENREF_3)]. Primary antibodies were obtained from Abcam: CD44 (ab157107), CD133 (ab226355), OCT4 (ab18976), SOX2 (ab97959), PD-L1 (ab201811), MRP1 (ab260038), P-gp (ab262880), PHF21B (ab119249) and β-actin (ab8226). All dilutions were 1:1,000.

**Dual-luciferase reporter assay**

The binding site of hsa_circ_0003222 and the 3′UTR of PHF21B, WT-circ_0003222, Mut-circ_0003222, WT-PHF21B -3′UTR, and MUT-PHF21B-3′UTR were inserted into the KpnI and HindIII sites of the pGL3 promoter vector (HanBio, Shanghai, China) in a dual-luciferase reporter assay. Cells were plated into 24-well plates. Then, 80 ng plasmid, 5 ng Renilla luciferase vector pRL-SV40, 50 nM miR-527 mimics, and negative control were transfected into cells with Lipofectamine 2000 (Invitrogen). The cells were then collected and measured 48 h after transfection using a Dual-Luciferase Assay (Promega, Madison, WI), following the manufacturer’s instructions.

**Sphere formation assay**

Cells were grown in MammoCult medium (Stem Cell Technologies, Vancouver, Canada) supplemented with MammoCult Proliferation Supplements (Stem Cell Technologies) and plated in 24-wells plates with ultra-low attachment at a density of 10,000 viable cells/mL and grown for 10 days. Spheres were counted and photographed.

**Animal studies**

To examine the role of hsa_circ_0003222 in a lung cancer metastasis model, the xenograft and the orthotopic experiments were performed with 6 mice in each group (control group, miR-527 inhibitor group, si-circ-2+mir527 inhibitor group, and si-circ-2 group). We injected 1×10^6^ stable si-hsa_circ_0003222-2 (si-circ-2) or miR-527 inhibitor PC-9 LCSCs intravenously through the tail vein into male nude mice (Chinese Science Academy, Shanghai, China). One month later, we measured and quantified the lung metastases by an in vivo bioluminescent imaging with an IVIS Lumina Series III in vivo Imaging System (PerkinElmer, New York, NY).

As for xenograft assays, we injected 1×10^6^ modified or control PC-9 LCSCs (control, si-circ-2, si-circ-2+miR-527 inhibitor, and miR-527 inhibitor PC-9 LCSCs) subcutaneously into the right side of each male nude mouse (n=6) (Chinese Science Academy). We measured the tumor volumes (length × width^2^ × 0.5) at specified time points. For the PD-L1 therapy study, we injected in the tail vein of the mice with a PD-L1 monoclonal IgG antibody (Bioss Inc, Beijing, China) or control at 100 μg per dose three times a week for two weeks. Tumors were excised 5 weeks after injection.

All mice were used BALB/c Nude, mice, 8 weeks old, 18-22g. All mice were randomly divided into each group.

**Histological detection**

Tumor tissue were fixed in 4% paraformaldehyde and embedded in paraffin. Sections were stained with Ki67 or Tunel to assess proliferationor apoptosis. Sections were detected via an Axiophot light microscope (Zeiss) and captured with a digital camera.

**Reference**

[1] Thomas MF, Ansel KM. Construction of small RNA cDNA libraries for deep sequencing. Methods in molecular biology. 2010;667:93-111.

[2] Liu W, Guo J, Luo J, Ren Q, Chen Z, Qu Z, et al. Analysis of microRNA expression profiles dynamic in different life stages of Haemaphysalis longicornis ticks by deep sequencing of small RNA libraries. Ticks and tick-borne diseases. 2020;11:101427.

[3] Zhang X, Hu F, Li C, Zheng X, Zhang B, Wang H, et al. OCT4&SOX2-specific cytotoxic T lymphocytes plus programmed cell death protein 1 inhibitor presented with synergistic effect on killing lung cancer stem-like cells in vitro and treating drug-resistant lung cancer mice in vivo. Journal of cellular physiology. 2019;234:6758-68.
