# Supplementary figures and images for: hsa_circ_0003222 accelerates stemness and progression of non-small cell lung cancer by sponging miR-527
Source: Cell Death Dis. 2021 Aug 25;12(9):807. doi: 10.1038/s41419-021-04095-8 (PMC8387484; doi:10.1038/s41419-021-04095-8)

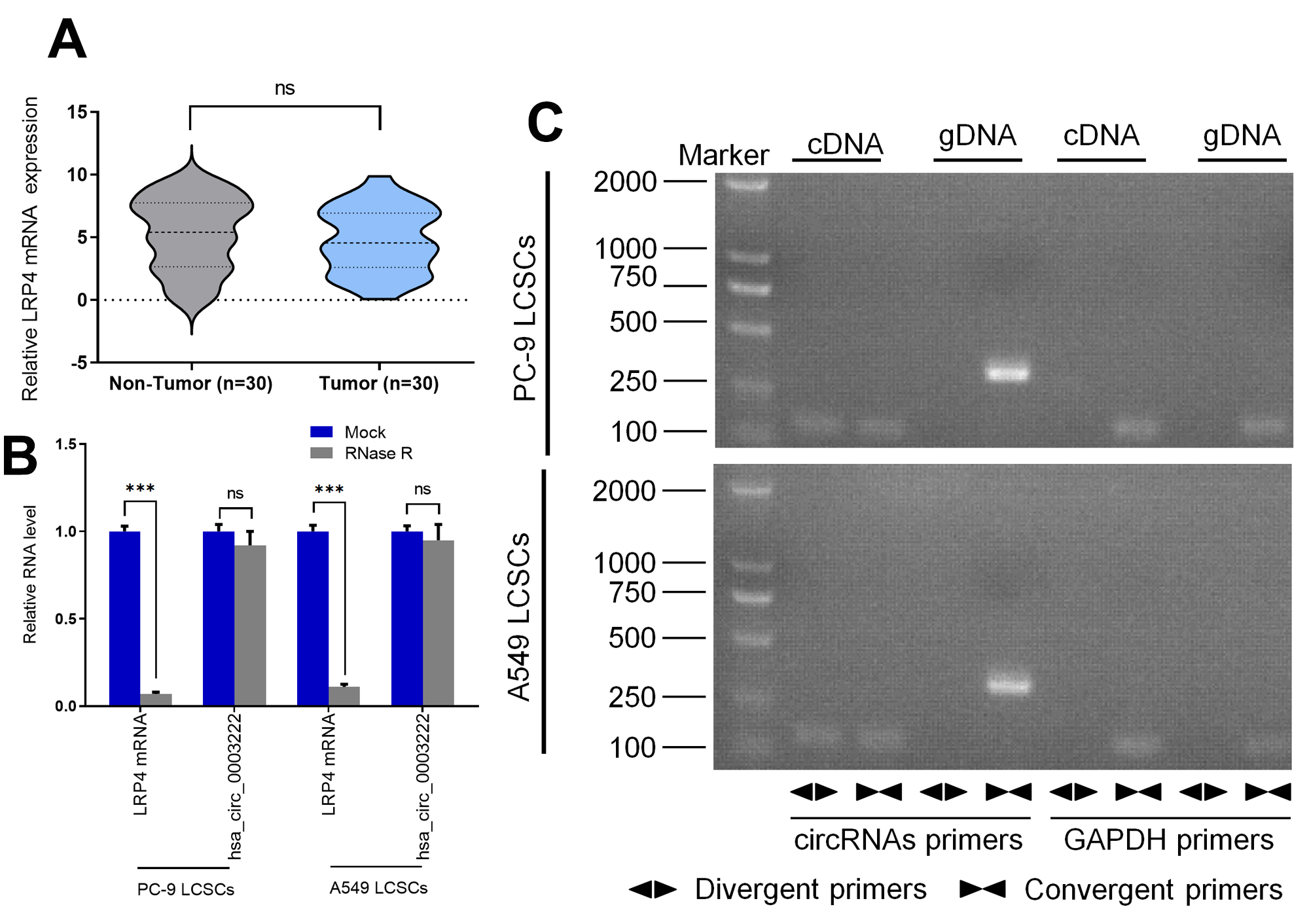

Supplement: Supplementary file 2 — supplement figure 1 [file 41419_2021_4095_MOESM2_ESM.tif]

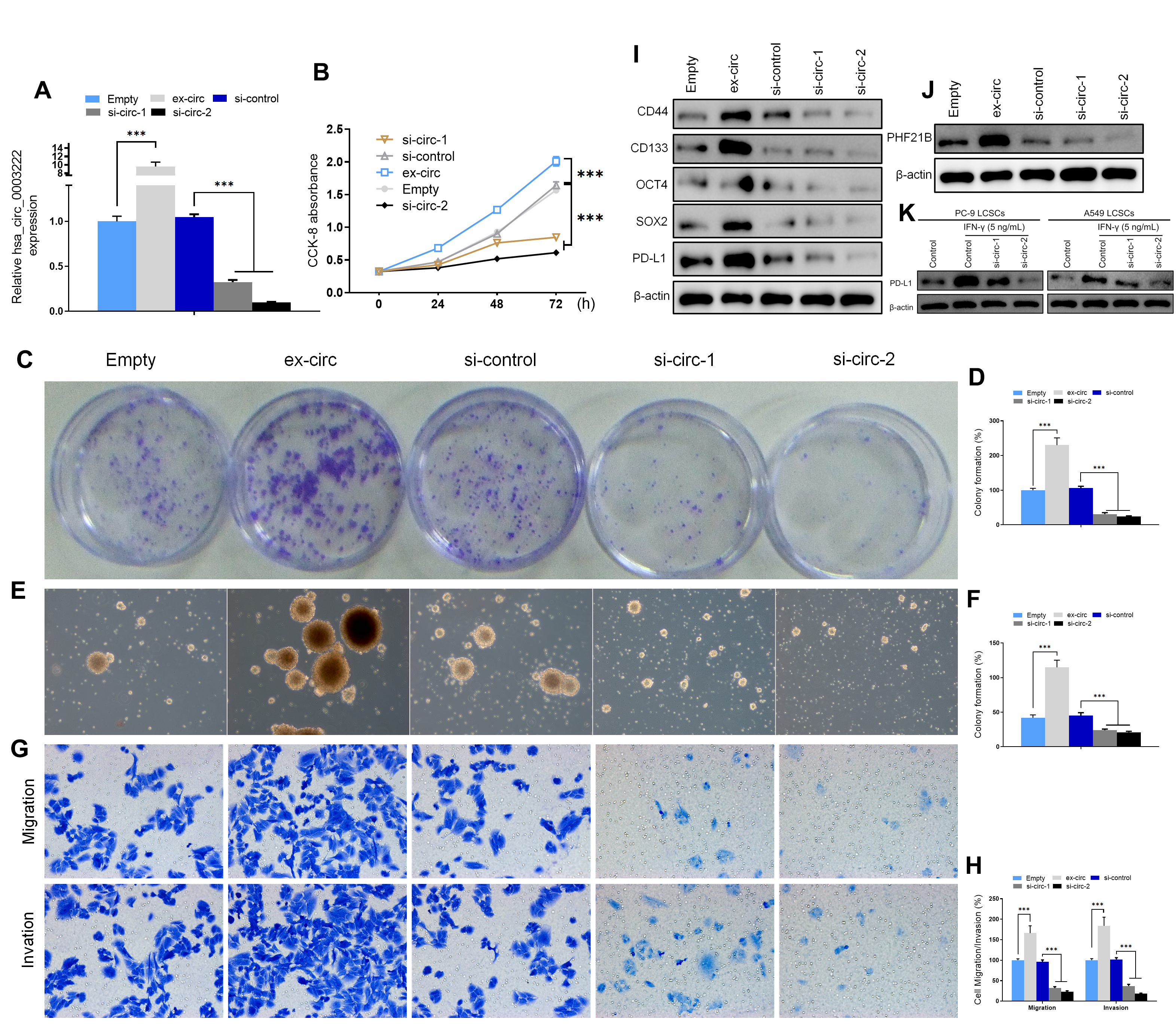

Supplement: Supplementary file 3 — supplement figure 2 [file 41419_2021_4095_MOESM3_ESM.tif]
